# Industrial Data Space Architecture Implementation Using FIWARE

**DOI:** 10.3390/s18072226

**Published:** 2018-07-11

**Authors:** Álvaro Alonso, Alejandro Pozo, José Manuel Cantera, Francisco de la Vega, Juan José Hierro

**Affiliations:** 1 Departamento de Ingeniería de Sistemas Telemáticos, Universidad Politécnica de Madrid, 28040 Madrid, Spain; apozo@dit.upm.es; 2 FIWARE Foundation, 10587 Berlin, Germany; josemanuel.cantera@fiware.org (J.M.C.); juanjose.hierro@fiware.org (J.J.H.); 3 School of Computer Science, Universidad Politécnica de Madrid, 28660 Boadilla del Monte, Spain; fdelavega@fi.upm.es

**Keywords:** industry 4.0, industrial data space, industry of things, internet of things, FIWARE

## Abstract

We are in front of a new digital revolution that will transform the way we understand and use services and infrastructures. One of the key factors of this revolution is related to the evolution of the Internet of Things (IoT). Connected sensors will be installed in cities and homes affecting the daily life of people and providing them new ways of performing their daily activities. However, this revolution will also affect business and industry bringing the IoT to the production processes in what is called Industry 4.0. Sensor-enabled manufacturing equipment will allow real time communication, smart diagnosis and autonomous decision making. In this scope, the Industrial Data Spaces (IDS) Association has created a Reference Architecture model that aims to provide a common frame for designing and deploying Industry IoT infrastructures. In this paper, we present an implementation of such Reference Architecture based on FIWARE open source software components (Generic Enablers). We validate the proposed architecture by deploying and testing it in a real industry use case that tries to improve the maintenance and operation of milling machines. We conclude that the FIWARE-based IDS implementation fits the requirements of the IDS Reference Architecture providing open source software suitable to any Industry 4.0 environment.

## 1. Introduction

We are in front of a new digital revolution which will transform the way we understand and use services and infrastructures. This revolution will affect the daily life of people and businesses and is centered in three main pillars: the e-business, the Web 2.0 and the Smart Life. E-business [1] refers to the presence of business (both Business-to-client and Business-to-business) on Internet, specifically to the new activities and practices created as a result of the business incursion in the IT world. Web 2.0 [2] is understood as the new Web concept created several years ago in which users are not just information consumers. In this new concept, users also enrich the Web with information they provide. Finally, Smart Life [3] is related to improving the daily life of people by means of the Internet of Things and smart devices. The most known use case is that of smart cities [4], but this also includes connected homes [5] and vehicles [6].

The new digital life will gravitate around the notion of context, which describes what is happening, where, when, how, etc. Thanks to this context, services and applications can access and manage information in several scopes. Thanks to recent research advances, context information is accessible by any kind of web service or application [7]. Cities are one of the most prominent examples of the importance of context. Context information about public services (transport, parks, streetlights, etc.), restaurants or even citizens creates a digital continuum, blurring the frontiers between application domains and facilitating the citizens daily life. On the other hand, the recent digitalization of production processes and the proliferation of the Industrial Internet of Things [8] bring this new digital paradigm to the scope of the industry and are part of the key elements behind Industry 4.0. Sensor-enabled manufacturing equipment allows real time communication, smart diagnosis and autonomous decision making.

However, beyond proprietary communication channels and centralized commercial marketplaces for data such as BDEX (http://www.bigdataexchange.com/), there is currently no standardized, widely accepted means for a trustful exchange of industrial data that ensures traceability, data owners’ privacy and sovereignty. In this scope, the Industrial Data Space initiative [9] creates a virtual data space for a reliable data exchange among industrial partners and tries to standardize the way in which data is managed, transported and secured in this kind of scenario.

Industrial Data Space was created in a research project funded by the German Federal Ministry of Education and Research (BMBF) involving multiple Fraunhofer institutes. As a result of this initiative, the Industrial Data Space Association (IDSA) (http://industrialdataspace.org/en) was created—an open, non-profit organization promoting the continuous development, exploitation and sustainability of the Industrial Data Space.

In the literature, we can find several works that propose approaches to deploy and manage Internet of Things (IoT) systems in the scope of Industry 4.0. They are focused on how to improve performance or to automatize production processes; how to secure exposed data or how to efficiently manage a huge amount of produced data. However, a common reference architecture to cover all these aspects and enable a global model for Industry 4.0 did not exist until the proposal of the Industrial Data Space.

Although the Industrial Data Space research project is focused on designing the Reference Architecture Model, the specific implementation of the architectural layers proposed in such a model are currently out of the scope of the reference. Furthermore, at the time of writing this manuscript, there is no open source implementation of those layers. Our contribution in this paper is the first open source implementation of the IDS Reference Architecture providing a set of software components that can be deployed in any Industry 4.0 scenario to include the architectural layers defined by IDS. The implementation includes the required protocols and modules to provide trusted and secure data sharing between industry entities and is based on FIWARE Generic Enablers.

The FIWARE European project (https://www.fiware.org/) aims to provide a framework for developing smart applications for the Future Internet. FIWARE is based on a service ecosystem composed of key elements called Generic Enablers (GEs), which encompass a framework that allows the development of smart applications relying on smart services and data management components.

FIWARE Generic Enablers are grouped into three main categories. The first is related to Context Management and involves software components based on publish/subscribe interfaces and Big Data analysis. The second involves Open Data and API management components and the last is focused on Identity and Access Control management. The documentation explaining how to install, configure and deploy FIWARE Generic Enablers can be found in the FIWARE Developers Catalogue (https://www.fiware.org/developers/catalogue/).

Besides the implementation, in this paper we describe a Zero-defects/breakdown manufacturing use case that validates the proposed implementation by materializing it in a real Smart Industry scenario.

The document is structured as follows. In the next Section, we provide an overview of the related research about IDS reference architecture. Section 3 introduces the main IDS Architecture concepts and how it is structured in layers. Then, in Section 4, we describe the solution we propose. Section 5 illustrates the scenario we have setup and developed to validate the proposed implementation. Finally, in Section 6, we summarize our work and explain the possible future lines of research.

## 2. Related Work

The research presented in this paper is principally based on the Industrial Data Space Documents [9,10]. They describe the whole Architecture Model for an industry oriented realization of a business ecosystem. Furthermore, it is focused on the security requirements that need to be addressed in order to ensure data sovereignty and privacy. Moreover, in [11] the authors describe the Information Model as well as the vocabulary used in the Reference Architecture Model of the Industrial Data Space [10]. The next section presents a detailed analysis of the proposed IDS architecture and how to implement it using FIWARE.

Industry has shown special interest in adopting IoT solutions in order to enhance performance of their systems. Xu et al. [12] analyze some IoT technologies that could be used in several industrial areas, such as logistics or mining. Furthermore, several challenges that should be addressed in coming years are described. The work remarks the necessity of designing an architecture that takes into consideration, among other factors, energy consumption reduction and performance enhancement. The authors also point out the necessity of creating standards for IoT with regards to security, identity management and communications. Finally, they also refer to the relevance of the security issue and why trust and reliability mechanisms should be improved in order to ensure data privacy.

The paradigm of Industry 4.0 brings new challenges regarding the automatization of IoT networks in order to perform information exchange in a timely, reliable and uniform way. New trends in industrial communication described in [13] as Ethernet time-sensitive networking (TSN) and Fifth Generation Wireless Systems (5G) consider the importance of this fact among other factors. An example of these factors is the management of heterogeneity due to the inclusion of end users who indirectly interact with IoT industrial networks following the “maker culture” concept.

Besides communication technology, the management of IoT networks is also crucial. [14] proposes an approach based on Group-based Industrial Wireless Sensor Networks (GIWSNs) and sleep scheduling of sensors. This achieves an improvement of the energy consumption and an easier deployment of IoT devices.

In [15], a software-defined architecture is analyzed for industrial scopes in which IoT systems would be deployed. In addition, several issues related to this architecture are taken into consideration. Regarding the security issue, authors enumerate several possible vulnerabilities and how they could be mitigated through Software Defined Networks (SDN). On the other hand, they underline the importance of creating standards for the interaction between all different components of an Industrial Internet of Things Architecture.

The inclusion of IoT systems in the Industry has involved the generation of a large amount of sensitive data and, consequently, an increase of cyber attacks. [16] shows some of the industrial IoT systems’ vulnerabilities and provides an overview of possible solutions around the architecture, integrity verification and device management of cyber-physical systems. This work also outlines the need to improve security mechanisms taking into account the heterogeneity of IoT networks.

In order to enhance flexible access control and authorization mechanisms for Industrial IoT systems, [17] proposes an authorization framework for large-scale scenarios based on metadata definition.

The large amount of data generated in Industry 4.0 implies the development of new schemes to transform them in valuable information. Yan et al. [18] outline the benefits of using Big Data towards Industry. In particular, authors propose a framework to structure and analyze industrial data, apart from describing some data mining techniques. Additionally, they prove its usability in a real use case study for predictive maintenance in a Computer Numerical Control Machining Center. Additionally, [19] praises the benefits of applying Digital Twin concepts along with Big Data analysis. Digital Twin could play an important role when designing IoT networks, through the mapping of physical devices and their behaviors to virtual schemes.

Fortino et al. [20] stress the importance of defining what is an IoT Service especially for large scale industrial scenarios. They propose a definition based on four properties: dynamicity (IoT services can be dynamically created), context-awareness (any information about IoT entities is relevant), co-location (several IoT services could share same physical resources) and transitivity (define when an IoT Service could end).

Finally, [21] explains some of the security considerations to be taken into account when deploying an IoT solution in a cement plant, which could be extrapolated to other areas of the Industry. For instance, firmware updates of IoT devices should be done regularly. They also specify Quality of Service (QoS) rules in order to guarantee the performance of security systems. These concerns are tackled when deploying the IDS architecture through FIWARE as described by next section.

As outlined in this section, we can find several works that propose approaches to deploy and manage IoT systems in the scope of Industry 4.0. Table 1 summarizes the main concepts in which the studied-related researches are based. As we can see, most of them are focused in the design of an architecture or in the study of the security issues of each specific scenario. On the other hand, the IDS Reference Architecture aims to provide a common architectural model to facilitate businesses to deploy them securely and efficiently. Furthermore, the IDS enables all these systems to interoperate so that they are capable of sharing data and bringing the industrial data management to the limit.

## 3. Industrial Data Space Concepts

As described in the Industrial Data Space documents [9,10], the Architecture Model is divided into five layers.

### 3.1. Business Layer

This layer refers to the high level architecture of the Industrial Data Space and establishes the basis for the rest of layers. It describes the participants that take part in the Architecture, their main activities from a business point of view and the interactions between all of them. Depending on the activity, the participants are grouped into four categories: Core Participants, Intermediaries, Software and Services and Governance Body.

### 3.2. Functional Layer

This layer describes all the necessary functional requirements of the Industrial Data Space Architecture as well as the needed characteristics. All these requirements are organized into: connectors, vocabulary and metadata management, app ecosystem, clearing house and identity management. Furthermore, trust and security enclose such functional entities and affect how their requirements are defined. Connectors are the key entity of this layer. They are used to exchange data between data consumers and data providers.

### 3.3. Process Layer

The process layer defines how the different participants communicate with each other in the Industrial Data Space Architecture. Three main processes are described in this layer: how to provide data, how to publish and use Data Apps and how to exchange data between data consumers and data providers.

### 3.4. Information Layer

The information layer provides an Information Model that the participants and components of the Industrial Data Space Architecture must adhere to in order to improve communication and integration. The Industrial Data Space Vocabulary provides models to identify and describe resources in the Industrial Data Space Architecture. Furthermore, an information model is provided to describe the components defined in the functional and business layers and the relationships between them.

### 3.5. System Layer

The system layer describes the architecture of a connector, which can be external (for data exchanging) or internal (providing data to external connectors). This structure, which is based on containers technology, can be divided into execution and configuration phases.

## 4. Materializing Industrial Data Space Architecture with FIWARE

This section describes the different components and perspectives the IDS Architecture model considers and how they have been implemented using FIWARE.

The description of the implementation is focused in the first two layers. Referring to the business layer briefly commented before, the components implemented are: App Store, Broker Provider, Identity Provider, Data Consumer and Data Provider. These two last components are responsible for the management of the connector, which is described in the functional layer and is composed by several systems adapters provided by the App Store. The Identity Provider will be described in the security architecture, which has to be considered in all layers.

### 4.1. System Adapter

IDS System Adapters play the role of mediators (or wrappers) between the original data sources (enterprise systems, IoT Service Layer components such as OPC-UA Servers, plain CSV files, etc.) and IDS Connectors. IDS System Adapters are in charge of adding extra metadata, for instance, contextual information such as temporal or geographical properties.

In FIWARE, a System Adapter is implemented by the conjunction of three components: IoT Agents (https://fiware-iot-stack.readthedocs.io/), Orion Context Broker (https://fiware-orion.readthedocs.io/) and OPC-UA IoT Agent (https://github.com/is3labengrd/iotagent-opcua).

The IoT Agent plays the role of IDS System Adapters in FIWARE-based IDS Architectures. In fact, IoT Agents act as adapters between the IoT Service and the Information Management Layer incarnated by IDS Connectors, i.e., the Orion Context Broker. There are different IoT Agents offered, but the most relevant one to the Industry 4.0 domain is the OPC-UA IoT Agent.

OPC-UA [22] is an standard for industrial communication that defines a protocol, an information model and the associated services related to the digitization of industries. OPC-UA is one of the many sources of context information that can be exploited by FIWARE. In fact, FIWARE offers harmonized APIs and information models that enable data sharing in multi-sided markets where IDS participants belonging to different smart domains (cities, industry, agriculture, ports) intervene and collaborate towards a common objective. For instance, manufacturing information exported by a factory through OPC UA and published to FIWARE via the NGSI protocol could be used by a local port to predict and plan future transportation needs.

OPC-UA offers several advantages from its predecessor (Open Platform Communications) that benefits the integration in Industrial scenarios [23]. Specifically, OPC-UA IoT Agent is capable of map between the objects exposed by an OPC-UA Server and the information model defined by NGSI (Next Generation Services Interface) [24], i.e., objects are mapped to entities, attributes and metadata.

### 4.2. Connector

**Figure 1:**
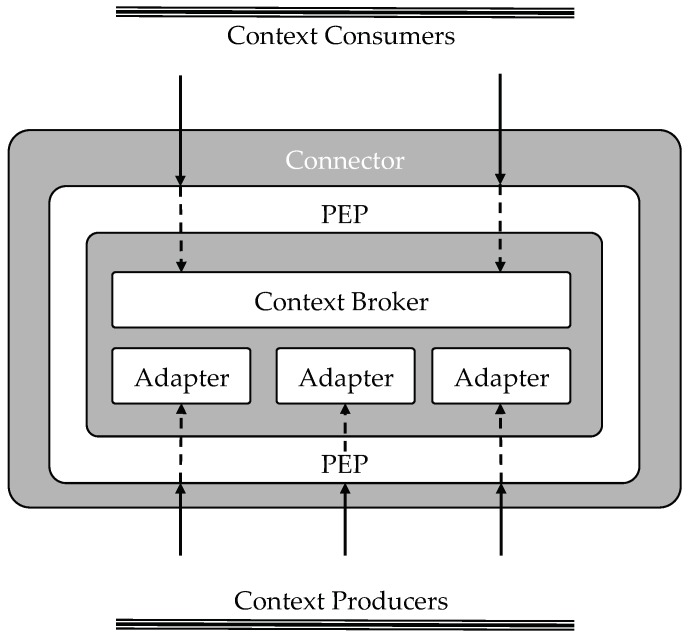
FIWARE IDS Connector.

The IDS Connector is responsible for the execution of the complete data exchange process. The Connector thus works as an interface between the internal data sources and enterprise systems of the participating organization and the Industrial Data Space. Figure 1 illustrates the components that compose a Connector. In terms of FIWARE Generic Enablers, a Connector is composed by an instance of the Orion Context Broker, a set of System Adapters and an instance of the Policy Enforcement Point (explained with detail in Section 4.5).

The Context Broker (Orion) is the core component of the IDS Connector in FIWARE-based implementations of the IDS Architecture. In fact, the Context Broker offers the FIWARE NGSI APIs and associated information model (entity, attribute, metadata) as the main interface for sharing data by the IDS participants. The combination of well-defined domain-specific data models and a harmonized API (NGSI), enables Data Producers and Consumers to participate seamlessly in the IDS. Essentially, Data Producers use NGSI to publish or to expose the data they offer (normally through a System Adaptor) and Data Consumers retrieve or subscribe (to be later notified) to the data offered. The operations concerning Data publication, consumption, subscription and notification are performed through the NGSI API.

To protect the access to the Connector (both for publications and subscriptions) a Poliy Enforcement Point (PEP) is included in the Connector. Thus, every request is intercepted by this component that checks if it is authorized to access the specified resource. Section 4.5 describes with detail the Identity and Access Control architecture.

### 4.3. AppStore

The IDS AppStore is responsible for the management of verified Data Services, which provide data transformation, curation, and processing to the IDS connectors (e.g IDS System Adapters). The IDS AppStore allows to publish, search, discover, acquire and deploy Data Services.

In the FIWARE-based implementation, the IDS AppStore is implemented by the Business API Ecosystem (https://business-api-ecosystem.readthedocs.io/) (a.k.a BAE), which provides the means for the publication and monetization of digital assets under different pricing models, managing also the usage terms and conditions. The particular Data Services offered through the BAE are hold as MasterMind (https://mastermind-main.readthedocs.io/) services. This tool is a management platform for FIWARE data and IoT services, which supports the automatic deployment of such components using Docker Swarm (https://docs.docker.com/engine/swarm/).

The access to the services managed by MasterMind is controlled using roles and permissions (see Section 4.5). In this regard, the BAE is the component in charge of granting those permissions when data services are acquired, so the customers are authorized to deploy them within their IDS Connectors.

### 4.4. Broker

The IDS architecture enables Data Providers to share their data with other participants (Data Consumers). Available data sources and its characteristics can be discovered by Data Consumers using an IDS Broker. In particular, an IDS Broker stores metadata which describes the data source, including information about the Data Provider, the syntax and semantics of the data, or additional information such as the pricing or the usage policies.

In the FIWARE reference implementation of the IDS Architecture, the data source metadata is described using an NGSI Registration. This format enables to provide information about an NGSI data source, including the endpoint, available data, or geographic location. In addition, it allows to include custom metadata, which can be used to provide usage policies and pricing information.

In the FIWARE-based IDS, the IDS Broker role is performed by an NGSI Registry, which provides the NGSI API for the management of NGSI Registrations, enabling to register, discover or subscribe them. In addition, the FIWARE-based IDS provides a data portal linked to the NGSI Registry which simplifies the discovery and registration of NGSI data sources. This data portal is implemented using a CKAN (https://ckan.org/) portal enhanced with a set of FIWARE CKAN extensions (https://fiware-ckan-extensions.readthedocs.io/).

Finally, the FIWARE-based IDS supports the monetization of the published NGSI data sources by integrating the data portal of the IDS Broker with the BAE. In this regard, this architecture allows to create data offers linked to the different NGSI Registrations, providing the tools for the establishment of agreements between Data Providers and Data Consumers. The BAE delegates on the Security Layer the access control features (see Section 4.5). Therefore, when access to a data source is acquired, the BAE grants permissions to the Data Consumer according to the existing access policies.

### 4.5. Security Architecture

A strategic requirement of the Industrial Data Space is to ensure a high level of protection and trust when exchanging data between participants. The Security Architecture provides means to identify participants, protect data communication and control data usage. The security aspects to take into account for ensuring this protection and trust affect all the architectural layers: business, functional, process, information and system. Furthermore, IDS take into account three main principles with regards to security.

- Reliable technologies: the aim of IDS Security Architecture is not to provide a new solution for problems already solved but to reuse and combine existing approaches bridging gaps when necessary.
- Scalable approaches: IDS does not enforce a single level of security to be applied for all participants. However, certain minimum security requirements have to be met by every participant and third parties have to be able prove them.
- Security pays off: Data Sources can mandate a certain set of security features to be fulfilled. Thus, a higher security level enables access to Data Sources of higher quality and to services of higher value.

The Security IDS Architecture addresses several security aspects that fit the exposed principles and ensures the protection of every architectural layer. These aspects are materialized in a Trusted Connector, a component constructed on top of the IDS Base Connector that adds the needed features to achieve the security architecture.

Bellow we briefly introduce the security aspects defined by IDS. After the analysis of each aspect, we describe the strategies and implementations we use to achieve them using FIWARE. The Generic Enablers that enable the introduction of the security aspects in the architecture are the Identity Manager-Keyrock (https://catalogue.fiware.org/enablers/identity-management-keyrock), the Policy Administration and Decision Point-AuthZForce (https://catalogue.fiware.org/enablers/authorization-pdp-authzforce) and the Policy Enforcement Point-Wilma (https://catalogue.fiware.org/enablers/pep-proxy-wilma). These components are explained with detail in the next paragraphs.

#### 4.5.1. Secure Communication

To ensure confidentiality and authenticity of the data transmitted between two participants, communication must be encrypted. Thus, transactions are protected against eavesdropping, manipulation and impersonation. IDS defines two layers of security with regards to communication between Connectors: point-to-point encryption using an encrypted tunnel and end-to-end authenticity and authorization.

As explained before, in our implementation communication and data exchange between Connectors relies on NGSI. This means the use of HTTP requests for managing the entire lifecycle of context information, including updates, queries, registrations, and subscriptions. The requests can be sent over the Internet or via a Virtual Private Network (VPN) depending on the specific scenario. In both cases, HTTPS, the secure version of HTTP protocol, is used. HTTPS [25] uses an added encryption layer of SSL/TLS to protect the HTTP traffic. Therefore, the point-to-point encryption is covered by this protocol.

On the other hand, FIWARE implements authentication and authorization by means of an Identity and Access Control model based on the OAuth 2.0 and XACML protocols. This model is explained in detail in the following paragraphs.

#### 4.5.2. Identity Management and Access Control

According to the IDS Architecture Model, Identity and Access Management (IAM) is mandatory in order to make access control related decisions based on reliable identities and properties of the participants. This includes identification (i.e., claiming an identity), authentication (i.e., verifying the identity) and authorization (i.e., making access decisions based on an identity). Every participant may possess attributes apart from its identity, and these attributes could vary dynamically. Thus, Connectors regulate access to data basing on different criteria: the specific identity of Connectors, Connectors attributes or security profile requirements.

**Figure 2:**
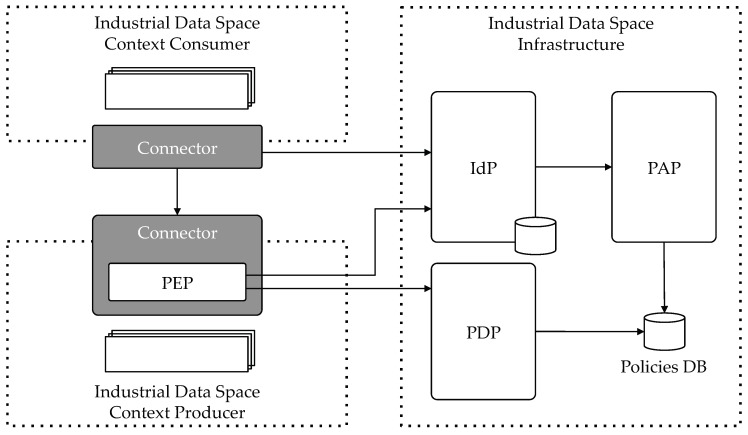
FIWARE IDS IAM Architecture.

Figure 2 shows the architecture we propose to achieve the Identity and Access Management IDS requirements using FIWARE components. It is based on the work explained in [26,27], where a generic IoT Application-Scoped Access Control as a Service (IAACaaS) mechanism is proposed. Here we extend and adapt it to fit the specific requirements of IDS. IAACaaS uses OAuth 2.0 protocol as authorization framework [28]. Using OAuth 2.0 delegates the authorization process saving load in IoT devices and enables the application-scoped feature. Thanks to this feature, Access Control policies can be defined in the scope of an application/service. By fostering this behavior, a participant could have different permissions for different services, thus making it possible for the same participant to be reused among different services, being under different security conditions in each of them.

In the Figure is depicted an IDS Context Consumer that accesses data provided by an IDS Context Producer. Of course, the communication between them is established through their respective IDS Connectors and using a secure channel. For protecting the access to the provided resources, every request is intercepted by a Policy Enforcement Point (PEP). On the other hand, in the IDS global infrastructure are deployed the rest of components that take part in the architecture. These components are deployed once and used by every IDS Connector in the environment. The Policy Administration Point (PAP) and the Policy Decision Point (PDP), together with the set of PEPs included in each Connector compose the widely-known Access Control architecture [29]. The PAP stores the defined access control policies in the Policies DB, where PDP checks them at decision time. Finally, the Identity Provider (IdP) is in charge of identification and authentication.

In order for the Access Control architecture to be used in all security contexts, the framework for describing authorization policies should fit a level of granularity and flexibility. For this purpose, OASIS (a global nonprofit consortium that works on the standards definition for security, IoT and other areas) standardized the eXtensible Access Control Markup Language (XACML) [30], which allows the definition of fine-grained policies. XACML serves as a standard not only for the format of authorization policies and evaluation logic, but also for that of the request/response interactions that take place during an authorization decision.

Following the XACML terminology, policies are composed by a set of rules (as well as other items that are out of the scope of this paper). Rules are in turn made of a target (e.g., the resource), an effect (e.g., allow/deny) and a condition. In our approach, rules are used to implement permissions, in which the target is an action (e.g., a HTTP verb) plus a resource of the Context Producer. Of course, more complex policies may also be defined, provided that they are supported by the policy-description language. Roles are in turn sets of Permissions that serve as a container so that more than one permission can be assigned at once. Both Permissions, Roles and their relationships are defined in the PAP. Note that this approach allows both a simpler Role-Based Access Control (RBAC) scheme and a more complex, more flexible Attribute-Based Access Control (ABAC) scheme. It all depends on how the Permissions are defined when creating the XACML rules.

As introduced above, the Identity Provider offers identification and authentication following an Identity as a Service (IDaaS) approach [31,32]. Thus, every IDS participant needs to be registered with the IdP, so that it gets a set of credentials (usually a username and a password), which can be used to authenticate and identify itself against the AC system. Groups of participants can be created in the IdP, to allow more complex authorization scenarios. Granting a permission to a group of participants, rather than to a single one, has the benefit of linking the given permission to the participants belonging to the group.

Once a participant is registered with the IdP, it can create an OAuth 2.0 access token for accessing data in an specific Context Producer. In OAuth 2.0 terminology, that means creating a token in the scope of a consumer. This token represents the participant in the system and has to be included in every request sent to the other Connector. As outlined before, these requests are intercepted by a PEP, that extracts the participant’s access token and validates it with the IdP. This validation can be performed in three levels of security:

1. Authentication: using this level of security, the PEP just checks if the participant has been correctly authenticated against the IdP. Thus, at this level, every participant with an active account would be able to access the protected data. The check is performed by sending a validation request to the IdP.
2. Basic authorization: in this case, the PEP also checks if the participant has the required roles to perform the corresponding action (defined by a HTTP verb) in the corresponding data source (defined by a HTTP path). After the first check with the IdP, the PEP obtains the roles the participant has assigned in the scope of the Context Producer where the token was created. Once roles have been retrieved, the authorization check is sent to the PDP. PDP fetches the policies associated with the participant’s roles from the Policies DB and decides whether or not access should be granted based on them.
3. Advanced authorization: this is the most complex, powerful case, because the authorization check is not only based on the HTTP verb and path, but also on other more advanced, customizable parameters, such as the request body or headers. To perform the check, a custom XACML policy request is sent to the PDP.

**Figure 3:**
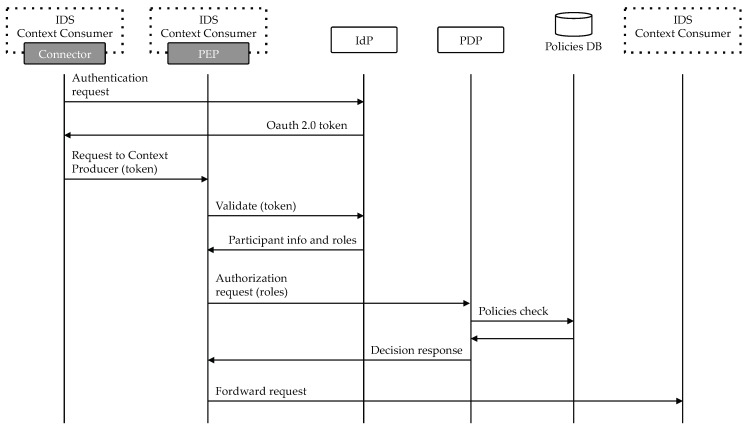
Authorization flow.

Figure 3 illustrates the interaction described below. Every component present in this architecture is implemented as a FIWARE Generic Enabler:

- Identity Provider: the Identity Management GE is named KeyRock and has been implemented using Node.js technology and npm libraries. Keyrock’s core is an OAuth 2.0 server but it also implements several extra functionalities to enable groups management, IoT devices registration and Access Control.
- Policy Administration and Decision Points: this implementation integrates both PAP and PDP components and is called AuthZforce. It provides an API to get authorization decisions based on authorization policies and authorization requests from PEPs. The API follows the REST architectural style and complies with XACML v3.0.
- Policy Enforcement Point: the policy enforcement point GE is named Wilma and is developed using Node.js. In the context of IDS FIWARE Architecture, Wilma is deployed as part of API Umbrella, an API Management system that enriches the PEP functionalities with features like accounting, API documentation or catching.

#### 4.5.3. Trust Management

Establishing trust between participants in the Industrial Data Space is crucial when talking about Security IDS Architecture. It happens that some Context Providers offer data that is not directly managed by them (i.e., stored in their databases) but provided by a third party Context Provider. In such cases, a participant could be authorized to get this data from the first Context Provider but not from the second one. The second provider, when receiving the delegated request, has to be able to authorize the request basing on the permissions the participant has in the scope of the original provider. For allowing this interaction, a trusted relationship has to be established between both Context Providers.

**Figure 4:**
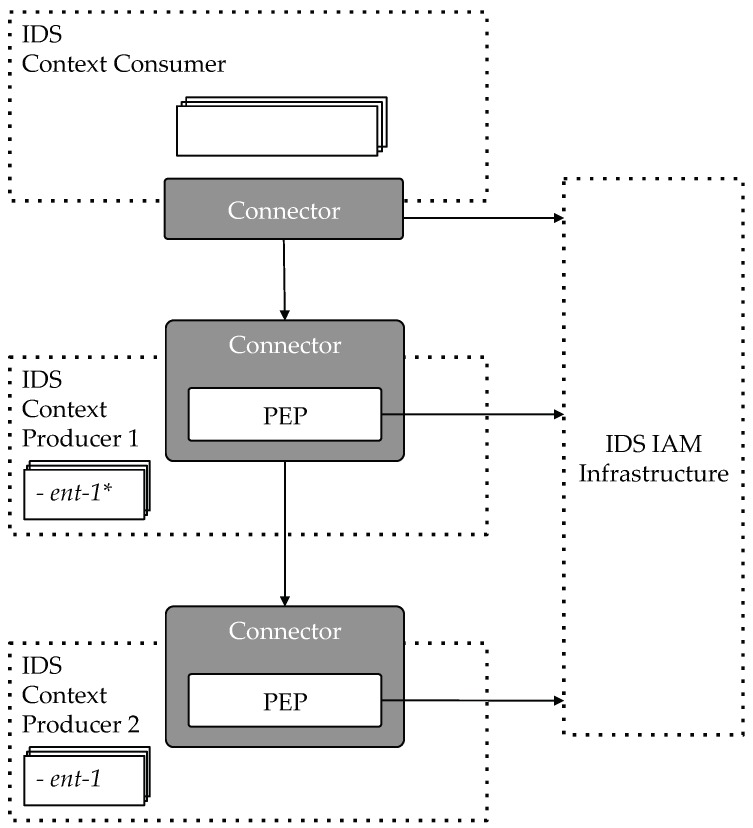
Trust between Context Producers.

Figure 4 illustrates the scenario. The Context Consumer has the needed permissions to access a data entity provided by Context Producer 1 (ent-1). However, although Context Producer 1 is offering this entity, it is actually provided by Context producer 2 so it has to delegate the Context Consumer request to it. When receiving the request, PEP of Context Producer 2 will check with the IAM infrastructure whether the consumer has the needed permissions to access such entity in the scope of the Context Producer 1. In this particular case, it should reject the request.

This issue is solved by FIWARE with a mechanism that allows a Context Producer to have a list of Trusted Context Sources that act as delegated Context Producers. When receiving a request from a participant, the PEP will check whether it has the required permissions to get access to data in the scope of itself or in the scope of one of the Context Sources included in its Truster Context Sources list. In the previous example, Context Producer 1 has to be included in the Context Producer 2 list. This way, when receiving the request it will check that having the participant no permissions defined in its scope, it actually has the permission to access the entity in the scope of a trusted Context Producer.

## 5. Use Case: Zero-Defects/Breakdown Manufacturing

One of the main applications of IoT in Industry is related to the predictive maintenance of production machines for improving their performance and quality [18,33]. For validating the solution described by this paper, we have implemented the proposed FIWARE architecture in a real use case: Zero-defects/breakdown manufacturing. It is a use case in which the manufacturing process of a factory is improved by analysing the data retrieved from their systems. Particularly, we take advantage of the data retrieved from two different kind of machines:

- Milling machines, producing objects by means of using rotary cutters to remove material from a workpiece of raw material.
- Coordinate-Measuring Machines (CMM), for measuring the physical geometrical characteristics of manufactured objects in order to detect defects.

Each machine could improve its configuration and maintenance by means of using data produced by the other machine, bringing overall a better service to the Factory. Data produced by the CMM could be useful for improving predictive maintenance of the milling machine (e.g., identify that the milling machine is about to start producing relevant defects so that maintenance tasks could be better planned). On the other hand, data produced by the milling machine could be useful for improving CMM recommended actions (e.g., what has to be reconfigured in the milling machine to sustain the quality of manufactured components).

In this use case, the Factory requires to get it certified that (1) only certain data is used by each machine manufacturer (2) such data is used only for declared purposes (e.g., improve maintenance) and (3) data from the company will not be disclosed to other competitors by the manufacturer of the machine.

Besides, machine manufacturers want to get it certified that only data they license under certain terms and conditions will be used by machine manufacturers which are capable to certify they will comply with the defined terms and conditions.

Taking advantage of the IDS architecture it can ensured that both parts of the system interchange and use data with the warranty of complying with their security and privacy concerns.

### 5.1. Deployment Set-Up

A pilot has been deployed, implementing the referred use case in which data is retrieved from two real machines in a factory: a Milling machine and a CMM machine. Both machines send data to an IDS Connector deployed by the factory. These data are consumed by two different services. The *Predictive Maintenance System* performs data analysis for predicting when the Milling machine needs a maintenance procedure. On the other hand, the *Quality Control* service studies the measurement data to evaluate the quality of production processes. Both services can subscribe to the data produced by each machine through two new IDS Connectors deployed in their respective infrastructure.

For performing data analysis, big data mechanisms could be used. In the designed pilot, after receiving the subscribed data, the services just show the data in a display. The experiment illustrates how configuring the right permissions and establishing a trust relationship between the connectors, the data can flow in a secure way between the participants.

**Figure 5:**
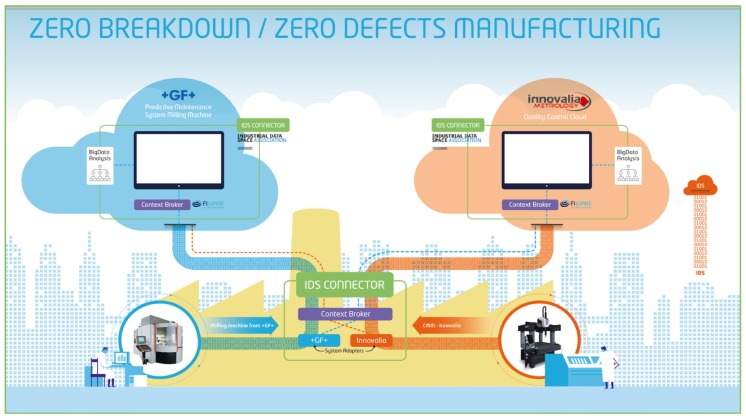
Use case set-up.

**Figure 6:**
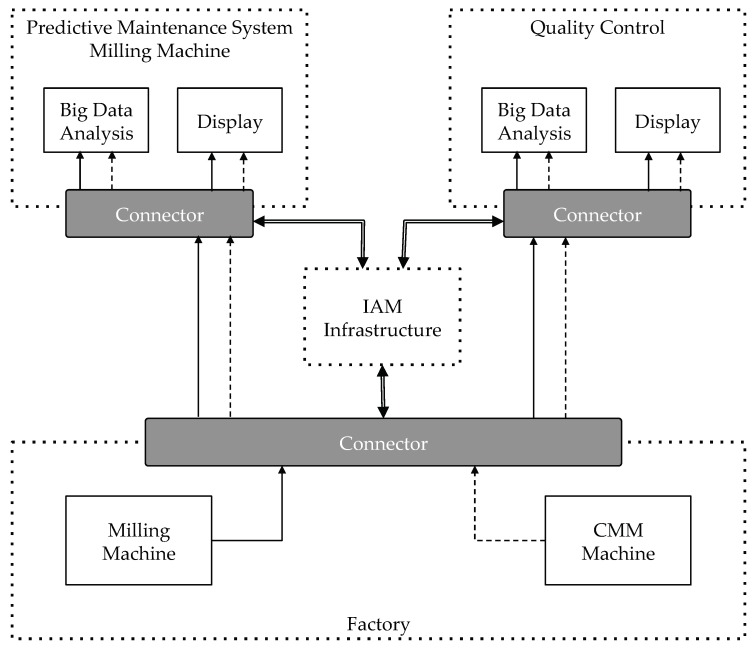
Use case modules and interaction.

Figure 5 shows how we set-up the pilot for displaying the data in the monitors. On the other hand, in Figure 6 the conceptual modules of the pilot can be shown.

In the factory shop floor, the Connector includes the needed System Adapters to convert the data produced by the machines to the format that the IDS Connector accepts. As depicted by the figure above, every Connector communicates with the central IAM infrastructure to validate that incoming requests are correctly authenticated and authorized to perform the needed actions. For simplifying the experiment, we have defined just two roles, *data-producer* and *data-consumer*. A participant with the role *data-producer* can publish data in the Context Broker of the Connectors. On the other hand, an entity with the role *data-consumer* can subscribe to the published data. Obviously, we have assigned the role *data-producer* to the machines and the role *data-consumer* to the applications that display the data in the monitors.

### 5.2. Results

**Figure 7:**
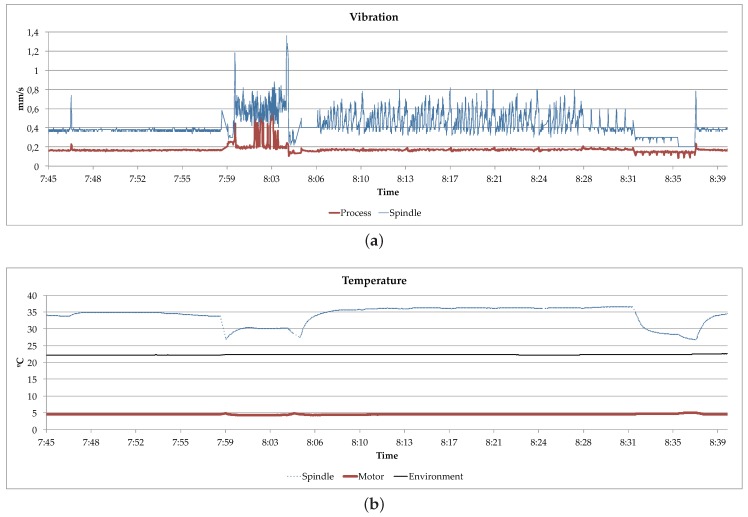
Milling Machine Predictive Maintenance Display. (**a**) Milling Machine Vibration; (**b**) Milling Machine Temperature.

**Figure 8:**
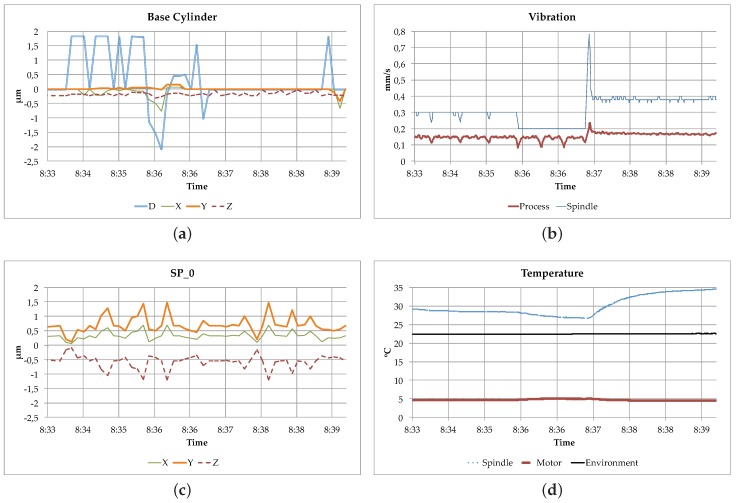
Quality Control Display. (**a**) Coordinate Measuring Machine Base Cylinder; (**b**) Milling Machine Vibration; (**c**) Coordinate Measuring Machine SP_0; (**d**) Milling Machine Temperature.

Figure 7 and Figure 8 show an excerpt of the data showed on the *Predictive Maintenance System* and the *Quality Control* displays when running the designed experiment. The *Predictive Maintenance System* uses the data received from the Milling Machine for monitoring the evolution of the temperature and the vibration. In addition, as there is also a subscription to the data produced by the CMM Machine, it could also get access to it if needed. On the other hand, the *Quality Control* service displays the received input from both machines, also the measurements produced by the CMM Machine.

In terms of Access Control management, the IAM infrastructure’s versatility when assigning and removing security policies plays a very important role. It is very common that the factory which owns the Milling and the CMM machines needs to preserve the privacy of the data. Thanks to the proposed security architecture, we address the requirement of the data sovereignty of the Industrial Data Space Architecture.

As explained before, the designed prototype shows how the data can be exchanged between the different participants according to the principles proposed by the IDS Reference Model. However, the data consumers (in this case the *Predictive Maintenance System* of the Milling Machine and the *Quality Control* service) are just displaying the data in their monitors. Therefore, no real Big Data analysis is performed and consequently no decision is taken accordingly, both things are out of the scope of this paper.

The proposed pilot was exposed as part of the FIWARE stand in the Industry Exhibition Hannover Messe, in April 2018 (https://www.fiware.org/news/fiware-at-the-hannover-messe-2018/) with more than 200.000 assistants and the feedback received from them was very positive.

When deploying the scenario in a production environment, the Big Data modules are the responsible of analyzing the received input and producing conclusions. On the one hand, the *Quality Control* service emits information reports about the quality of the produced objects. On the other hand, the *Predictive Maintenance System* estimates when the Milling Machine requires a maintenance action depending on the status of the machine and on the quality reports.

## 6. Conclusions and Future Work

As explained before, there are several approaches that solve some of the requirements to be addressed in Industry 4.0, for instance the securitization of IoT networks or the use of Big Data techniques to obtain valuable information from the data generated by sensors. The IDS Reference Architecture does not only provide a versatile and heterogeneous model, which allows the integration of new functionalities, but also takes into consideration security aspects to be addressed in industrial environments.

In this paper, we have proposed an implementation of the Industrial Data Space Reference Architecture. The implementation fits the security and interoperability requirements of the IDS Reference Model and its materialized by means of FIWARE Generic Enablers. Due to the open source nature of these components, we provide an implementation that can be easily adopted by any Industry environment. Furthermore, and thanks to the high flexibility of the software, the implementation can also be easily adapted to the specific requirements of diverse use cases.

To prove this, we have deployed the proposed implementation in a real Industry 4.0 scenario that analyses data produced by a Milling Machine and a CMM System to perform predictive maintenance of machines and to detect defects in production processes.

The next steps are mainly related to the improvement of the implementation by achieving several recommendations that, not being mandatory to achieve the requirements of the model, will enhance its performance. For instance, and regarding the Security Architecture, the study of the Industrial Data Space Communication Protocol (IDSCP) [10] to encrypt data exchange between connectors using Secure Web Sockets (WSS).

On the other hand, new communications technologies such as TSN or 5G will generate a great impact in the automation of IoT networks. Consequently, how these new technologies could fit within the Industrial Data Space Architecture should be studied in the future.

Finally, the deployment of the proposed elements in Cloud-based infrastructures would improve the scalability and the performance of the IDS systems. The application of already defined mechanisms for IoT [34] to the Industry 4.0 field should be explored in the future.

## Figures and Tables

**Table 1:** Related work comparison.

Reference	Architecture	Security	Big Data	Communications	Scalability
Wollschlaeger et al. [13]				X	
Lin et al. [14]	X				
Wan et al. [15]	X	X		X	
Sadegh et al. [16]		X			
Chen et al. [17]	X	X			X
Yan et al. [18]			X		
Qi et al. [19]			X		
Fortino et al. [20]	X				X
McNeil et al. [21]		X			
